# MF59-based lipid nanocarriers for paclitaxel delivery: optimization and anticancer evaluation

**DOI:** 10.1038/s41598-025-91504-z

**Published:** 2025-02-24

**Authors:** Marzieh Attar, Fatemeh Tash Shamsabadi, Alireza Soltani, Mohammad Taghi Joghataei, Seyed Reza khandoozi, Shahram Teimourian, Majid Shahbazi, Vahid Erfani-Moghadam

**Affiliations:** 1 Cellular and Molecular Research Center, Iran University of Medical Sciences (IUMS), Tehran, 1416634793 Iran; 2 Medical Cellular and Molecular Research Center, Golestan University of Medical Sciences (GOUMS), Gorgan, 4934174515 Iran; 3 Department of Medical Biotechnology, School of Advanced Technologies in Medical Sciences, Golestan University of Medical Sciences (GOUMS), Gorgan, 4934174515 Iran; 4 Golestan Rheumatology Research Center, Golestan University of Medical Sciences (GOUMS), Gorgan, Iran; 5 Cancer Research Center, Golestan University of Medical Sciences (GOUMS), Gorgan, Iran; 6 Department of Medical Genetics, School of Medicine, Iran University of Medical Sciences (IUMS), Tehran, 1416634793 Iran

**Keywords:** Cancer, Cytotoxicity, Drug delivery, Nano-carrier, Paclitaxel (PTX), NLC, Breast cancer, Cancer therapy, Cancer, Medical research, Molecular medicine, Oncology

## Abstract

Breast cancer is the most common invasive cancer in women worldwide, necessitating innovative therapeutic strategies to enhance treatment efficacy and safety. This study focuses on the development and optimization of novel paclitaxel (PTX)-loaded nanostructured lipid carriers (NLCs) that incorporate components of MF59, an oil-in-water emulsion adjuvant approved for use in influenza vaccines and known for its safety in humans. The formulation of these NLCs is designed to overcome significant challenges in PTX delivery, particularly its poor solubility and the side effects associated with traditional formulations containing Cremophor EL. We prepared two sets of NLC formulations using different liquid-to-solid lipid ratios through hot melt ultrasonication. Characterization of the selected formulations, NLC_Pre_ and NLC_Lec_, was conducted using dynamic light scattering (DLS), scanning electron microscopy (SEM), Fourier-transform infrared (FT-IR) spectroscopy, and ultraviolet-visible (UV-Vis) spectroscopy. The mean diameters were 120.6 ± 36.4 nm and 112 ± 41.7 nm, with encapsulation efficiencies (EE) of 85% and 82%, and drug loading (DL) of 4.25% and 4.1%, respectively for NLC_Pre_ and NLC_Lec_. In vitro cytotoxicity assays demonstrated that these MF59-based NLCs effectively target MCF-7 (Michigan Cancer Foundation) breast cancer cells while minimizing toxicity to normal HDF (human dermal fibroblasts) cells, thus enhancing the therapeutic index of PTX and offering promising clinical implications for breast cancer treatment.

## Introduction

Breast cancer represents the most commonly diagnosed malignancy among females worldwide, with an incidence rate that far surpasses other invasive cancers^1^. Recent estimates indicate that approximately 1 in 7 women, accounting for approximately 14% of the female population, will receive a breast cancer diagnosis during their lifetime^2^. The substantial burden this disease places on society highlights the urgent need for more clinical and molecular research. Evaluation of novel treatment modalities may help to better predict outcomes and improve management approaches for the millions of women affected by breast cancer annually. Such efforts are warranted given the substantial adverse impact of this malignancy in terms both of morbidity and associated healthcare costs^3,4^.

Paclitaxel (PTX) represents a potent anti-neoplastic compound with wide application in the management of various malignancies. Derived from taxane diterpenes isolated from the Pacific yew tree, PTX functions as a microtubule inhibitor to prevent cell division. Specifically, by promoting microtubule polymerization and stabilizing microtubule structure, PTX interferes with mitotic spindle formation, thereby inducing apoptosis in rapidly proliferating cancer cells. This mechanism of action, coupled with its well-established anti-tumor efficacy profile, have rendered PTX a mainstay in breast and other cancer chemotherapy regimens^5–7^.

Due to PTX’s extremely low aqueous solubility (0.1 µg/mL)^8^, commercial preparations contain high concentrations of Cremophor EL (polyethoxylated castor oil) as a solubilizing vehicle. However, Cremophor EL is linked to several serious adverse effects, including hypersensitivity reactions, gastrointestinal toxicity, and the induction of drug resistance. These toxicity issues posed by Cremophor EL significantly hamper the clinical intravenous delivery of PTX, limiting drug dosing and efficacy^9–15^.

Consequently, there is an urgent need to develop alternative nanoscale delivery platforms that can enhance PTX’s solubility profile while improving safety, to allow higher drug loading (DL) capacities and enable more effective intravenous anti-cancer chemotherapy through optimal pharmacokinetic performance. Overall, devising Cremophor EL-free formulations capable of optimized PTX solubilization remains an active area of investigation aimed at deriving clinical benefit from this otherwise highly potent tubulin-targeting anti-mitotic agent. Considerable research efforts have evaluated alternative PTX delivery platforms that avoid the use of Cremophor EL, including liposomes^16^, emulsions^17^, cyclodextrin^18^, microspheres^19^, mixed micelles^20^, and polymeric nanoparticles^21^. Specifically, various lipid nano-carriers such as nano-emulsions (NE), solid lipid nanoparticles (SLN), and nanostructured lipid carriers (NLC) have also been investigated for PTX delivery^22–24^.

Bangham introduced one of the earliest forms of nanostructured lipids in 1961, comprising spherical phospholipid vesicles ranging from tens to hundreds of nanometers in diameter^25–27^. Subsequently, NLCs were developed based on solid lipid nanoparticles, consisting of a lipid matrix incorporating both solid and liquid lipids encapsulated within an aqueous core surrounded by a lipid bilayer. Key advantages of NLCs as drug carriers include low toxicity, low immunogenicity, heightened stability, and biocompatibility coupled with the capacity to shield drugs from degradation while improving drug release (DR) kinetics. As an optimized lipid-based nano-carrier system, NLCs demonstrate promise for efficiently encapsulating and delivering therapeutic agents with enhanced safety profiles attributable to their tunable physicochemical properties^28–34^.

The objectives of the present study were to develop and characterize two novel PTX-loaded NLC formulations incorporating MF59 constituents (Squalene, Span 85, Tween 80) and solid lipids (Precirol, Lecithin) for assessment as potential drug delivery systems for breast cancer treatment. The formulation and characterization aimed to evaluate the encapsulation efficiency (EE) of PTX, examine the physicochemical properties of the NLCs, and assess their cytotoxic effects. Prior investigations have shown MF59 emulsion containing Squalene can reduce drug release (DR) rates, and increase drug solubility and bioavailability of lipophilic pharmaceutical agents^35^. We have previously investigated MF59-like formulations for gene delivery, which has provided us with valuable insights into the design and application of lipid-based carriers in therapeutic contexts^36^. In another study, we used MF59 and a MF59-based NLC for encapsulation and combinatorial effects of olive leaf extract (OLE) and oncolytic Newcastle disease virus (NDV) in cancer treatment. The results confirmed the ability of MF59 and MF59-based NLC for anti-cancer activity through a synergistic mechanism^37^.

This study builds upon previous works where MF59 nanoparticles efficiently encapsulated PTX and demonstrated delivery to MCF-7 breast cancer cells and HDF normal fibroblasts, achieving favorable encapsulation, DL, and particle characteristics^38^. Unlike our previous MF59 nano-emulsion, the NLCs developed here incorporate solid lipids (Precirol/Lecithin) to enhance drug retention and stability, addressing limitations in sustained release. Ultimately, this work seeks to evaluate the potential of biocompatible MF59-based NLCs as an effective drug carrier for PTX administration in breast cancer treatment, building upon the promising results previously achieved with MF59 nano-emulsion and we showed that NLCs provide a more efficient system for controlled DR when compared to MF59. We developed two novel PTX-loaded NLC compositions based on MF59 constituents, optimizing them for high drug encapsulation efficiency (EE), tunable particle characteristics, and controlled drug release (DR) profiles. Our physicochemical profiling demonstrated that these MF59-derived NLCs effectively encapsulated PTX while selectively targeting MCF-7 tumor cells.

## Materials and methods

### Materials

PTX (white powder with purity of 99.5%) was provided by Nanoalvand Pharmaceutical Company (Tehran, Iran). A vial of PTX solution (6 mg/mL) was purchased from Sobhan Darou Oncology Company (Tehran, Iran). Tween 80, Span 85 and Squalene were purchased from Sigma Company (USA). Water was purified using a Milli-Q Plus 185 water purification system (Millipore, USA).

For the in vitro and cell culture studies, Dulbecco’s Modified Eagle Medium (DMEM), penicillin/streptomycin mixture, trypsin-EDTA and fetal bovine serum (FBS) were purchased from Gibco Life Technologies (Carlsbad, CA, USA).

Precirol, Lecithin and 3-(4,5-dimethylthiazol-2-yl)−2,5-diphenyltetrazolium bromide (MTT) were obtained from Sigma-Aldrich (Saint Louis, MO, USA).

The MCF-7 breast cancer cell line and HDF normal cells were kindly supplied by the Medical Cellular and Molecular Research Center in Gorgan, Iran. All other reagents and materials were purchased from commercial suppliers without further purification.

### Preparation of NLCs

In this study, we investigated three liquid lipid to solid lipid weight ratios (50:50, 40:60, and 30:70) based on the components of MF59 for the manufacturing of NLC_Pre_ and NLC_Lec_. Utilizing a fixed total lipid mass of 200 mg, these ratios enabled us to evaluate the resulting nanoparticle properties, PTX EE, and subsequent toxicity into cells.

The NLC formulations were prepared using a modified hot melt ultrasonication method^37,39^.

Initially, the lipid phase, consisting of Squalene (liquid lipid) and Precirol or Lecithin (solid lipids), was melted at 61 °C in an incubator (Memmert, INE 400, Germany) until a homogeneous mixture was achieved. Concurrently, an aqueous phase containing Tween 80, Span 85, and citrate buffer (comprised of citric acid monohydrate and trisodium citrate dihydrate, pH: 6.5) was also heated to 61 °C. Following this, the hot aqueous phase was added to the molten lipid phase and further diluted with warm ultrapure water to reach a final volume of 10 mL (Table 1).

This precursor emulsion underwent ultra-sonication using a Misonix XL-2000 (USA) for three cycles of 30 s each at maximum power to obtain the desired NLC formulations. The mixture was then stirred for 10 min at room temperature. All NLCs were stored overnight at 4 °C to ensure their stability and uniform distribution prior to characterization and application.

The methodology employed in this study aligns with the approach utilized by SP de Sousa Marcial et al., who also employed hot melt homogenization followed by ultrasonication for the encapsulation of PTX within various lipid nanosystems^23^.

The hot melt ultrasonication method is favored for several reasons. Firstly, it allows for the production of NLCs with a consistent particle size, which is crucial for ensuring uniform drug release and bioavailability.Additionally, the hot melt ultra-sonication technique is advantageous due to its simplicity and reproducibility. This method is suitable for large scale production. Moreover, this method is known for its high EE, which is vital for maximizing the therapeutic potential of the loaded drug. It does not require the use of toxic organic solvents, making it a more environmentally friendly option compared to other methods^40,41^.

**Table 1:** Composition of the lipid nano-carriers.

Composition	Name					
	NLC_Pre_−50	NLC_Pre_−60	NLC_Pre_−70	NLC_Lec_−50	NLC_Lec_−60	NLC_Lec_−70
Oily phase						
Squalene	78.5 mg	63 mg	47 mg	78.5 mg	63 mg	47 mg
Precirol (Pre)	78.5 mg	94 mg	110 mg	-	-	-
Lecithin (Lec)				78.5 mg	94 mg	110 mg
Aqueous phase						
Tween 80	23 mg	23 mg	23 mg	23 mg	23 mg	23 mg
Span 85	20 mg	20 mg	20 mg	20 mg	20 mg	20 mg
Buffer citrate	1 ml	1 ml	1 ml	1 ml	1 ml	1 ml
Pure water	Upto 10 ml	Upto 10 ml	Upto 10 ml	Upto 10 ml	Upto 10 ml	Upto 10 ml

### Particle size, polydispersity index (PDI), and zeta potential

The average particle diameter and PDI of all formulations were assessed using dynamic light scattering (DLS) analysis. This was performed on a Microtrac® MRB particle size analyzer (NANOTRAC Wave II, Canada) at temperature of 25 °C. Microtrac® MRB is a highly flexible DLS analyzer which allows faster measurements with reliable technology, higher precision, and better accuracy^42^.

The zeta potential of nanoparticles were investigated to assess their surface charge characteristics, which are critical for understanding their stability in suspension^43^. Measurements were conducted at a controlled temperature of 25 °C using the Malvern Zetasizer (Malvern Instruments, UK). The Zetasizer is such an easy system to use for the analysis of low volumes of nanoparticles in various dispersants for size and zeta potential measurements^44^.

### PTX-loaded NLCs

The prepared nanostructured lipid carriers (NLCs) were evaluated to select formulations demonstrating optimal physicochemical properties for PTX encapsulation investigations. Based on characterization results including optimal particle size, zeta potential and good stability, NLC_Pre_−60 and NLC_Lec_−60 were chosen to prepare PTX-loaded NLC_Pre_ and PTX-loaded NLC_Lec_, respectively.

To incorporate PTX, 10 mg of the drug was initially dissolved in 500 µl absolute ethanol. This PTX-ethanol solution was then added directly to the molten lipid phase of NLC_Pre_−60 and NLC_Lec_−60, immediately prior to mixing the aqueous phase and applying ultra-sonication. A fixed lipid to drug mass ratio of 20:1 was maintained for both PTX-NLC formulations. The selection of this ratio and the inclusion of 10 mg of the drug were experimentally and based on our previous studies. This approach aimed to ensure an excess amount of the drug, thereby maximizing the nano-carriers encapsulation capacity.

All other formulation procedures for the PTX-NLCs were identical to the previously described NLC synthesis method. The selection of optimal non-drug NLC types for DL based on initial screening aided development of formulations with desirable encapsulation attributes.

### Encapsulation efficiency (EE) and drug loading (DL)

EE and DL of the nano-carriers were indirectly assessed via centrifugation as previously described^38^. Nano-emulsions were centrifuged at 6,000 rpm for 20 min, after which the precipitated drug was dissolved in isopropanol and drug concentration in the sediment was determined using UV-visible spectrophotometry at 227 nm and the standard calibration curve. EE and DL were calculated using the following formulas:

$${\text{EE}}\left( \% \right)=({\text{Drug entrapped in nano-carriers}}/{\text{Total drug added}}) \times 100\%$$

$$DL\left( \% \right)=\left( {{\text{Drug entrapped in the nano-carriers}}/{\text{Total lipids added}}} \right) \times 100\%$$

### In vitro drug release (DR) study

The release of PTX from NLCs was measured as previously described^38,45^. The ability of new formulations to modulate DR was evaluated by assessing cumulative PTX release from nano-carriers into an aquatic environment with a pH of about 7, over time via centrifugation method.

Briefly, the nano-emulsions, which were refrigerated to ensure their stability and uniform distribution, were centrifuged at 6,000 rpm for 20 min at various time points (2, 4, 9, 16, and 26 days post nanoparticle production) to separate the encapsulated PTX colloid as supernatant and then returned to storage. After dissolving the precipitate in isopropanol, the amount of released drug in the sediment was measured using UV spectrophotometry at 227 nm. Each nano-carrier was tested in triplicate.

### Scanning electron microscopy (SEM)

Nanoparticle size and morphology were evaluated using SEM. A Zeiss EVO 50 EP microscope (Zeiss, Oberkochen, Germany) operated at 15 kV was used to image the gold-coated nanoparticles. Coating with a thin gold layer preceded SEM visualization to render the specimens electrically conductive. This analytical technique provided high-resolution surface characterization of the formulated nanoparticles.

### FT-IR spectroscopy

Fourier transform infrared (FT-IR) spectroscopy was conducted to preliminarily evaluate potential physical and chemical interactions between PTX and the constitutive lipids of the nano-carriers. Pure PTX and lyophilized nano-carrier suspensions were homogenously mixed with spectral-grade KBr powder before condensing into discs for analysis. FT-IR scans provided information on the chemical structure and compatibility between drug and lipid components within the optimized nano-formulations.

### Ultraviolet-visible (UV-Vis) spectroscopy

Optical absorption of NLC_Pre_, NLC_Lec_, PTX-NLC_Pre_ and PTX-NLC_Lec_ was measured from 190 to 700 nm using a UV-Vis spectrophotometer (DeNovix DS-11, USA) at room temperature. Pure water served as blank reference for nano-carrier absorption profile. Wavelength scans were performed to generate absorption spectra of the samples using the instrument software. This analytical approach characterized the intrinsic optical properties of the developed nano-formulations.

### Cell culture

MCF-7 (human breast cancer) and HDF (human dermal fibroblast) cells were obtained from the Medical Cellular and Molecular Research Center, Gorgan, Iran. Cells were cultured at 37 °C in low glucose DMEM supplemented with 10% FBS, 2 mM L-glutamine, 0.01 mg/ml insulin and 1% penicillin/streptomycin. Cultures were maintained in a 5% CO2 humidified incubator (BINDER, Germany). This validated protocol established optimal cell line propagation conditions for subsequent cytoxicity experiments.

### Cytotoxicity assay and cell viability

The cytotoxicity of nano-carrier formulations was evaluated via MTT assay in MCF-7 breast cancer and HDF cells. The MTT assay measures metabolic activity via tetrazolium salt reduction to insoluble formazan by viable cells.

Cells were seeded at 10,000/well in 96-well plates with DMEM and incubated at 37 °C, 5% CO2 for 24 h. Media was replaced with freshly prepared treatment solutions of PTX-NLC_Pre_, NLC_Pre_, PTX-NLC_Lec_, NLC_Lec_ and free PTX (6.25–400 µg/mL PTX). Empty carriers used equal suspension volumes. Because of very low solubility of PTX in aqueous environments and DMEM medium (approximately 0.1 µg/mL), we had to use a commercial drug that contains Cremophor EL as the free drug.

After 24 h, MTT solution was added for 3 h, followed by DMSO to dissolve formazan. Absorbance was read at 570 nm to quantify viable cells versus untreated controls, performed with ≥ 3 replicates. Cytotoxicity profiles elucidated the dose-dependent effects of test formulations by capitalizing on cellular redox activity, as indirectly gauged through metabolic conversion of MTT’s tetrazolium salt. This standardized, high-throughput colorimetric assay effectively evaluated treatment bioactivity in both cancerous and non-cancer cell lines.

### Statistical analysis

Cytotoxicity analyses were verified using two-way Analysis of Variance (ANOVA) testing with GraphPad Prism 8.2.1 software at the 95% confidence interval. All measurements were conducted in at least three independent replicates. Data are presented as mean values ± standard deviation (SD). Differences were considered statistically significant for *p* < 0.05.

## Results and discussion

### Characterization of synthesized lipid nanoparticles

The mean particle size, PDI, and zeta potential of the nanoparticles were assessed using DLS analysis. Measurements were performed in triplicate and are reported as the average ± standard deviation. In this study, we investigated the zeta potential of nanoparticles to assess their surface charge characteristics, which are critical for understanding their stability in suspension.

As shown in the Table 2, zeta potential ranged from − 20.2 mV to −28.8 mV across formulations, likely attributable to variations in solid: liquid lipid ratios used in production. The more negative zeta potential values suggest a stronger electrostatic repulsion among the particles, which is crucial for preventing aggregation. This is particularly relevant given that our empirical observations of the nano-functional formulations over several months indicate a high degree of stability, with no significant sedimentation or aggregation observed.

PDI is a critical parameter in nano-carrier systems, as it reflects the uniformity of particle size distribution. A low PDI (typically < 0.3) indicates a more homogeneous population of nanoparticles, which is crucial for ensuring consistent drug release, predictable pharmacokinetics, and improved stability of the nano-carriers. This uniformity minimizes batch-to-batch variations and enhances the reproducibility of the drug delivery system.

Studies have shown that lipid-based nano-carriers with a PDI below 0.3 exhibit improved therapeutic performance due to enhanced stability and predictable drug release profiles^46^. Based on these considerations, the PDI values reported in our study indicate a well-controlled formulation suitable for drug delivery applications.

All nanoparticle mean sizes were below 200 nm, spanning 107–186 nm (Table 2). Interestingly, formulations containing higher solid lipid proportions tended to produce particles of smaller hydrodynamic size, with an inverse correlation observed between solid lipid content and mean diameter. While zeta potential values were negative, no clear relationship was evident for zeta potential. The inclusion of nonionic surfactant Tween 80 imparted steric stabilization to the nanoparticle surfaces through its ethoxylated groups, inhibiting aggregation. These results are consistent with previous studies evaluating similar lipid-based nanoparticles. For example, Marcial et al. and Bang et al. both reported producing PTX-loaded nanostructured lipid carriers with mean diameters under 200 nm and negative surface charges using comparable production methods involving hot melt emulsification and ultrasonication^23,47^. In other study, Priya Patel et al. were fabricated NLCs for brain targeting by using an ultra-sonication approach and different surfactants. The particle size and EE were 205 ± 0.87 nm and 75.21 ± 0.98% respectively^48^.

Based on the comprehensive characterization of the formulations, we selected NLC_Pre_−60 and NLC_Lec_−60 for the encapsulation of PTX due to their favorable physicochemical properties. Specifically, NLC_Pre_−60 exhibited an optimal particle size of approximately 120.6 nm, which is advantageous for enhancing cellular uptake and ensuring effective biodistribution.

Additionally, both formulations demonstrated a negative zeta potential, indicating good stability and reduced aggregation tendencies, which are critical for maintaining the integrity of the nanoparticle system. Stability assessments over a one-month period revealed no visible precipitation or color changes, further confirming the robustness of these formulations. The lipid composition at 60% was strategically chosen to balance drug loading capacity and the physicochemical characteristics necessary for effective drug delivery. This optimization is essential, as it influences the overall performance of the nanoparticles, ensuring that they meet the criteria for successful therapeutic applications.

A study conducted by Huguet-Casquero A. et al. was in line with our evaluation. In this study several solid–liquid lipid proportions ranging from 10:90 to 90:10 were used for constructing NLC to study their effect in formulation characteristic. Finally the formulation 60:40 was selected for the loading studies^39^.

**Table 2:** Physicochemical characterization of blank lipid nano-carriers.

Formulation	Diameter (nm)	SD	PDI	Zeta potential (mv)
NLC_Pre_-50	154.2	60.50	0.1689	−20.5
NLC_Pre_-60	120.6	36.4	0.254	−22.9
NLC_Pre_-70	114.7	61.7	0.614	−24.7
NLC_Lec_-50	186.1	68.80	0.0730	−20.2
NLC_Lec_-60	112	41.7	0.1158	−24.9
NLC_Lec_-70	107	35.80	0.1249	−28.8

### Evaluation of EE and DL

As previously described, the formulations selected based on characterization, NLC_Pre_−60 and NLC_Lec_−60, were loaded with PTX. The percent EE of PTX in PTX-NLC_Pre_ and PTX-NLC_Lec_ was determined to be 85% and 82%, respectively, using a centrifugation technique. Corresponding DL was 4.25% and 4.1%. No statistically significant differences were observed between the two NLC types regarding EE or DL (*P*> 0.05). These results corroborate our prior findings demonstrating high encapsulation of PTX within MF59 nanoparticles^38^. Comparing the three nano-carriers, MF59 showed the highest EE of 88%, while NLC_Lec_ achieved the lowest of 82%; however, no carrier significantly differed in EE or DL values (*P* > 0.05). Overall, both NLC formulations exhibited favorable EE and DL profiles comparable to MF59, suggesting suitability for further evaluations. Optimization of physicochemical properties through formulation is critical to encapsulation performance.

Some previous studies have had similar results to our evaluation. For example, Prajapati B. et al. fabricated drug-loaded NLC by solid (Precirol ATO 5) and liquid lipid (Miglyol 812) with %EE 76.56 ± 0.12 that showed prolonged release of medicament which may reduce dose or dose frequency^49^.

### Evaluation of in vitro DR kinetics

**Fig. 1:**
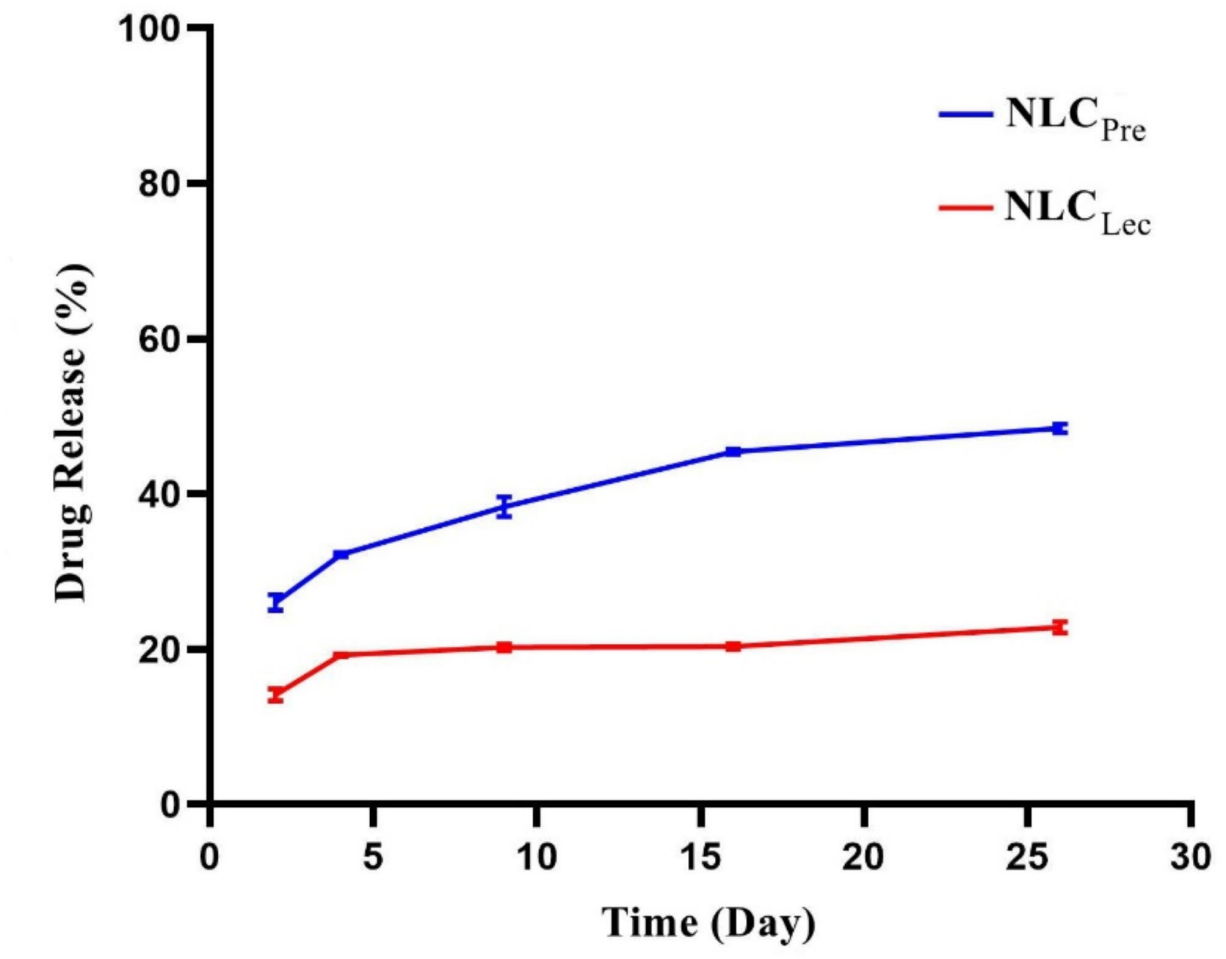
In vitro release percentage of PTX from NLC_Pre_ and NLC_Lec_.

DR from the nanoparticles was assessed in vitro over predetermined time intervals via centrifugation-based quantification. The percentage of PTX released was plotted versus time (Fig. 1), and NLC_Pre_ exhibited more rapid release kinetics compared to NLC_Lec_. Specifically, around 48% of PTX was released from NLC_Pre_ after 26 days, versus only 22% from NLC_Lec_over the same duration. A comparison of PTX release from NLCs and MF59 nanoparticles^38^ revealed slower release from the NLC systems. This suggests the poorly water-soluble drug was effectively encapsulated within the NLCs’ lipid cores, permitting gradual release over a prolongated period. Such a profile may confer clinical advantages through improved administration compared to MF59. While EEs were comparable, DR rates differed between carriers. Specifically, incorporating solid lipid into MF59 did not impact encapsulation but reduced the DR rate. Overall, the data implicate NLCs as nano-reservoir delivery platforms, slowly releasing PTX through retention within the particulate lipid matrix over extended timeframes. About of the difference in drug release efficiency between the two formulations, a possible explanation lies in the nature of the solid lipid used. This difference can be attributed to the physicochemical properties of Lecithin, including its amphiphilic nature and stronger molecular interactions with PTX. Lecithin consists of phospholipids that can form hydrogen bonds and hydrophobic interactions with the drug, potentially leading to a stronger retention within the nano-carrier matrix. In contrast, Precirol is a more hydrophobic lipid with a less interactive matrix, facilitating a slightly faster drug release. Additionally, differences in the lipid arrangement between the two formulations may influence the drug diffusion rate. Lecithin-based nano-carriers may form a more organized lipid matrix, restricting drug mobility, whereas Precirol-based carriers may create a less ordered structure, allowing for slightly higher drug diffusion.

In the study conducted by Gao et al., the   in vitro drug release of nab-paclitaxel was assessed using PBS supplemented with 0.05% (W/V) Tween 20 and 0.05% (W/V) Tween 80 via a dialysis method. The literature supports the assertion that the solubility of PTX is a critical determinant of its release dynamics. They demonstrated that the initial burst release and subsequent sustained release of PTX formulations were notably affected by the dialysis release method^50^. This finding is consistent with our results, where the centrifugation method effectively isolates unencapsulated drug, thereby providing a more accurate representation of the release profile over time.

This experimental design enabled the examination of time-dependent release profiles from the formulations over an extended period. Given the low solubility of PTX in aqueous environments (approximately 0.1 µg/mL)^8^, the use of a centrifuge method instead of a dialysis bag appears more appropriate. The dialysis method, while commonly employed, presents inherent contradictions; despite the use of surfactants such as Tween 20 and Tween 80 to facilitate drug diffusion, the presence of these surfactants does not accurately mimic physiological conditions where such agents are absent. Moreover, the dialysis release method acts as a diffusion barrier for poorly water-soluble drugs, which may lead to an unrealistic assessment of drug release kinetics^51^. Our centrifugation method avoids artificial surfactant use, providing a more accurate prediction of in vivo behavior where surfactants are absent.

Since lipid-based nano-carriers release their drug payload in vivo primarily through interactions with cell membranes rather than passive diffusion, there is no standardized in vitro method that fully mimics in vivo conditions. However, our approach provides a controlled assessment of drug release kinetics over an extended period. While the refrigerated storage condition differs from physiological temperature, it ensures the stability of the lipid nanoparticles throughout the study and prevents premature degradation or aggregation.

### Evaluation of nanoparticle morphology via SEM

SEM was used to assess nanoparticle morphology and conformational characteristics. SEM images revealed consistency between the morphology of drug-loaded and unloaded formulations, aligning with average particle sizes determined by DLS analysis. This similarity suggests that the incorporation of PTX did not significantly alter the structural integrity of the nanoparticles. From a stability perspective, maintaining a uniform morphology between loaded and unloaded nanoparticles is beneficial, as it indicates that drug encapsulation does not induce aggregation or structural deformation. This stability is crucial for ensuring consistent drug release kinetics, predictable pharmacokinetics, and prolonged circulation time in vivo.

**Fig. 2:**
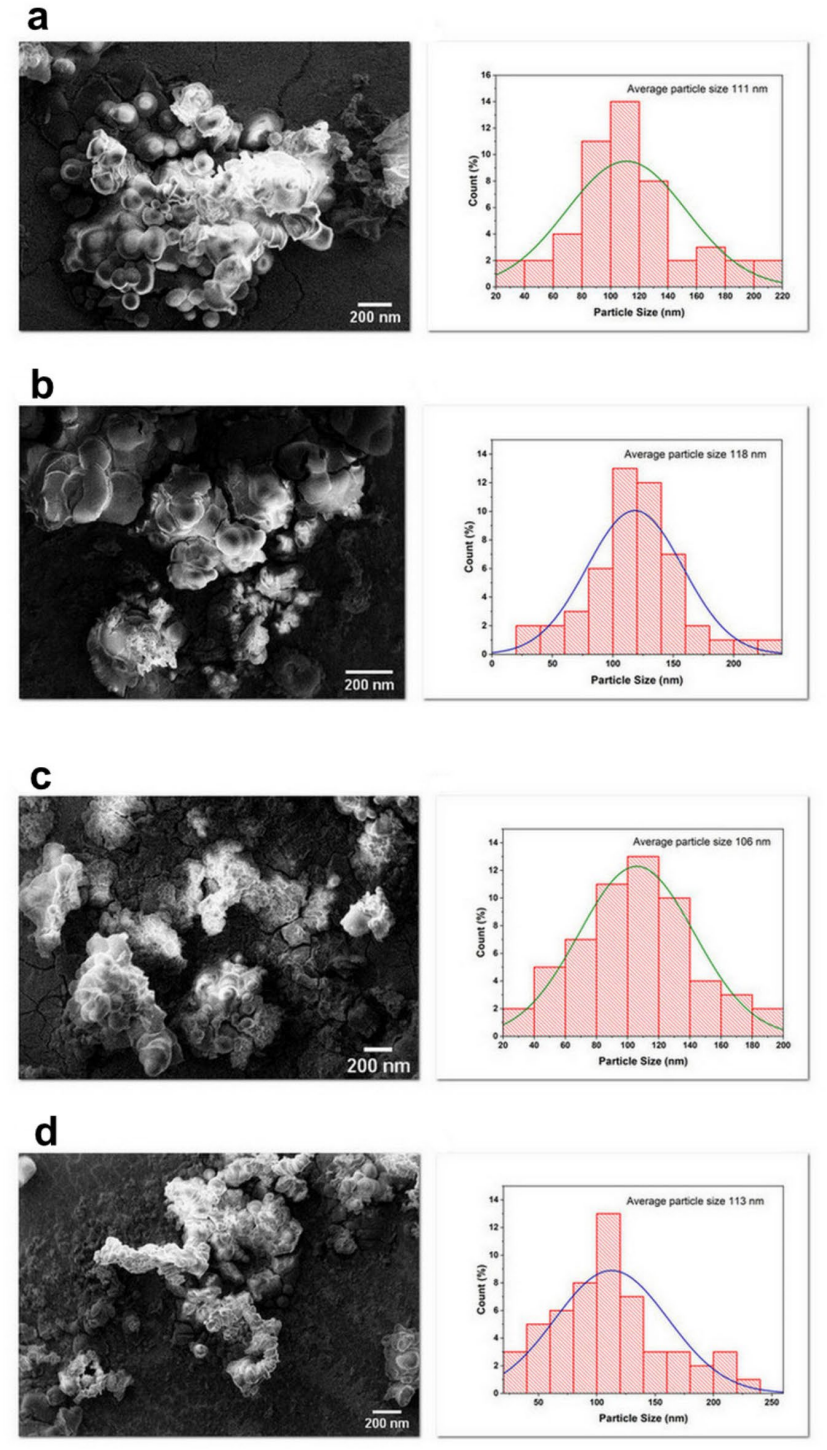
SEM images and particle size graph of the nanoparticles: (**a**) NLC_pre_, (**b**) PTX-NLC_pre_, (**c**) NLC_Lec_ and (**d**) PTX-NLC_Lec_.

As seen in Fig. 2, populations exhibiting diameters under 500 nm predominated across all formulations. Overall, SEM visualization corroborated spherical morphologies for the nanoparticles and corroborated size measurements obtained via DLS analysis. The evaluation suggests similar particle characteristics were maintained following drug encapsulation. Together, DLS data and SEM micrographs substantiate the production of monodisperse nanostructures in the 100–500 nm range. This characterization further validates the synthesis approach and selection of formulations for subsequent experimental applications based on uniform physicochemical properties.

It should be noted that some degree of nanoparticle aggregation may be observed in the SEM images compared to DLS size measurements. This is commonly seen and arises due to the processing required to prepare dried samples for visualization under SEM. Specifically, dropping a suspension onto a surface and allowing solvent evaporation can induce inter-particle interactions leading to the formation of aggregates. However, the consistent morphologies evident across drug-loaded and unloaded formulations, together with sizes aligning with DLS data, confirm that similar monodisperse nanoparticles were generated through the synthesis approach. The observation of some aggregate structures in SEM should therefore not detract from the validity of the overall particle characterization achieved through complementary DLS and SEM techniques.

### FT-IR

**Fig. 3:**
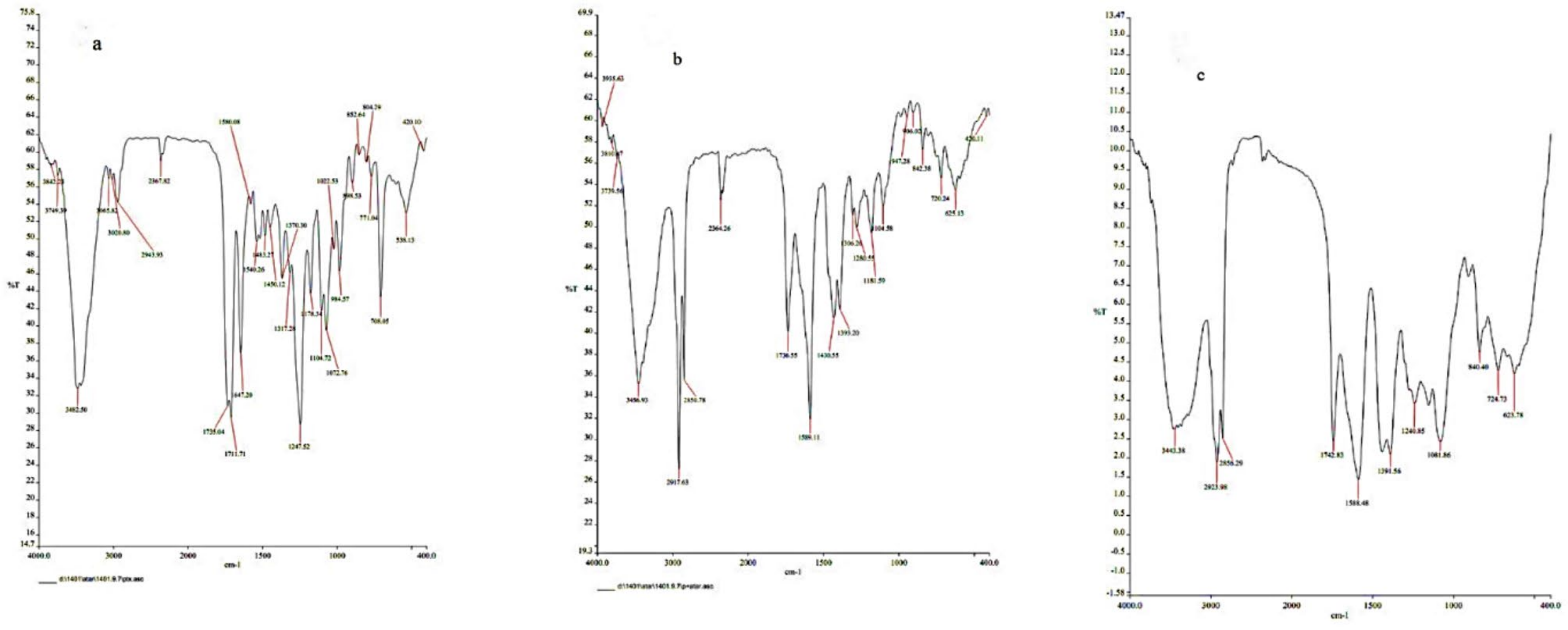
FT-IR spectra of (**a**) free PTX, (**b**) PTX-NLC_Pre_ and (**c**) PTX-NLC_Lec_. The peaks are discussed in the text and labeled in the figure.

The presence of distinct peaks was evaluated using Fourier-transform infrared (FT-IR) spectroscopy, with spectra collected using a Perkin Elmer Spectrum RX1 spectrometer over a wavenumber range of 400–4000 cm^−1^ for PTX-NLC_Pre_ and PTX-NLC_Lec_ nanoparticles as well as free PTX (Fig. 3). The spectra of free PTX and MF59 have been discussed previously^38^. In the spectrum of free PTX, peaks in the range of 3000–3200 cm^−1^ correspond to C–H stretching of aromatic groups. Peaks from 2900 to 3000 cm^−1^ are characteristic of C–H stretching of aliphatic groups. A peak at 1733 cm^−1^ represents carbonyl (C = O) stretching from the phenyl group, while a peak at 1711 cm^−1^ corresponds to the carboxyl group (C = O) from COOH. A peak at 3482 cm^−1^ indicates the presence of the PTX hydroxyl group. For PTX-NLC_PRE_, a peak at 1736 cm^−1^ represents carbonyl (C = O) stretching from PTX. A peak at 3456 cm^−1^ is specific to the hydroxyl group of PTX in the complex. Peaks at 2850 and 2917 cm^−1^ correspond to the C–H stretching of aliphatic groups, while a peak at 1589 cm^−1^ is characteristic of the C–C stretching of aromatic groups. A peak at 1104 cm^−1^ represents ether (C-O) stretching. The FT-IR spectrum of PTX-NLC_LEC_ exhibited peaks at 2856 and 2923 cm^−1^ characteristic of aliphatic C–H stretching. A peak at 3443 cm^−1^ corresponds to hydroxyl groups. A peak at 1081 cm^−1^ is indicative of C–O stretching of ether groups. A peak at 1742 cm^−1^ represents carbonyl (C = O) stretching, while a peak at 1588 cm^−1^ represents C = C stretching.

### Ultraviolet-visible spectroscopic analysis

**Fig. 4:**
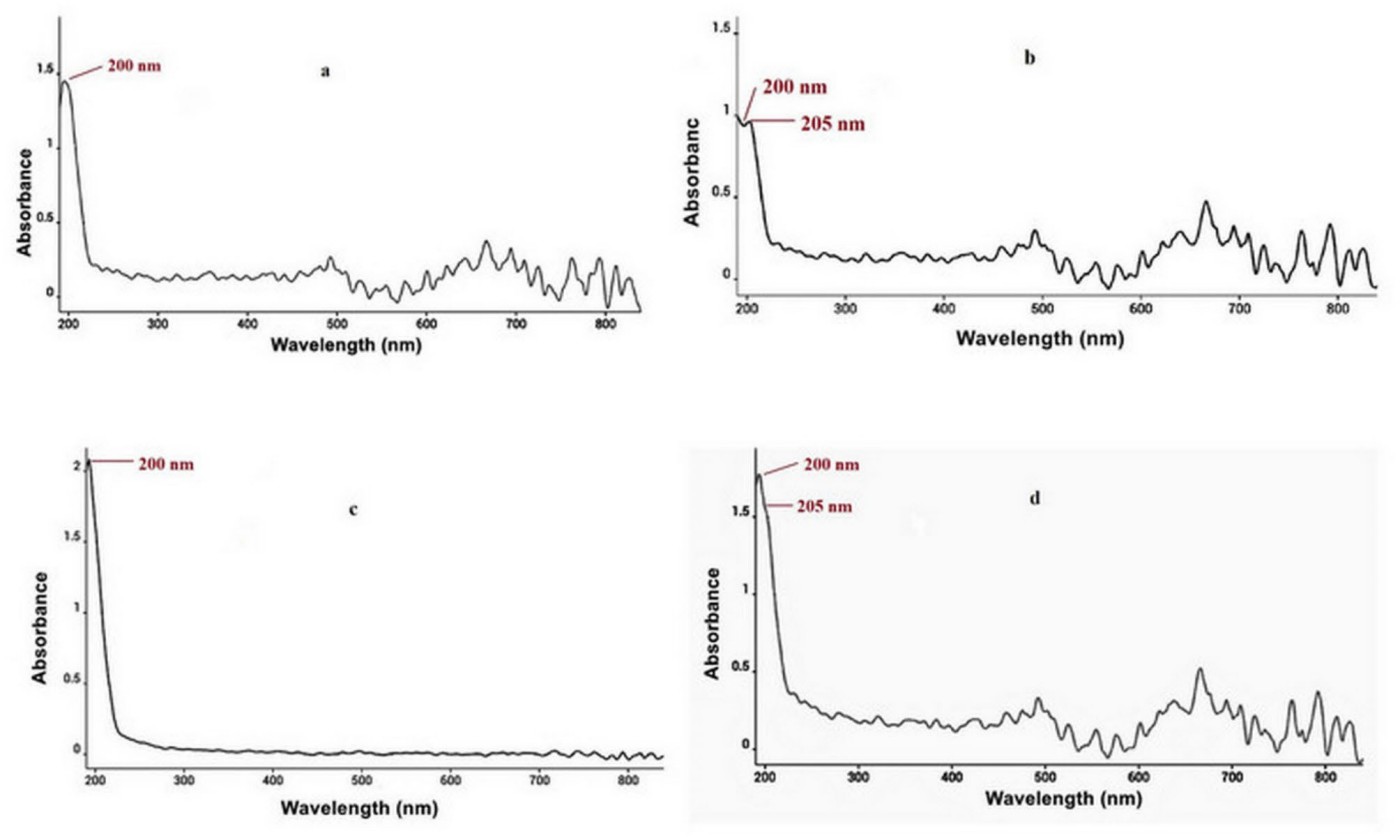
UV-Vis spectra of nanoparticles: (**a**) NLC_Pre_ (**b**) PTX-NLC_Pre_ (**c**) NLC_Lec_ and (**d**) PTX-NLC_Lec_.

Ultraviolet-visible (UV-Vis) absorption spectra of NLC_Pre_, NLC_Lec_, PTX-NLC_Pre_, and PTX-NLC_Lec_ are presented in Fig. 4. Distinct absorbance peaks were observed at 205 nm attributed to π-π electronic transitions within the conjugated systems of the nanoparticles and PTX. Encapsulation of PTX produced a redshift of this peak to 220 nm. This spectral shift provides evidence of molecular interactions between drug and carrier, such as hydrogen bonding, that facilitate intramolecular charge transfer (π-π interactions). Comparable observations have been reported previously for PTX-MF59 complex^38^. Taken together, the UV-Vis data corroborate the successful encapsulation of PTX within the particulate systems at the molecular level, as evidenced by distinctive absorbance signatures and a diagnostic peak displacement. Overall, the spectroscopic analysis substantiates the formation of drug-nanocarrier and provides insight into intermolecular forces governing encapsulation interactions. This characterization complements the physicochemical profiling of the PTX-loaded formulations.

The FT-IR and UV-Vis data provide evidence for successful encapsulation, but a detailed analysis of the spectral changes is necessary to understand their implications for drug stability and release. In the FT-IR spectra, shifts in characteristic PTX peaks (e.g., C = O, O-H, and aromatic ring stretches) compared to free PTX will be quantitatively analyzed. These shifts will be interpreted to determine the nature and strength of interactions between PTX and the NLC lipid components (hydrogen bonding, van der Waals forces, and hydrophobic interactions). Such interactions can significantly influence PTX stability by mitigating hydrolytic degradation or oxidation. The extent of these protective effects will be assessed by comparing the stability of PTX within NLCs to that of free PTX under accelerated conditions. Furthermore, changes in the UV-Vis spectrum, such as shifts in λmax or changes in absorbance intensity will be correlated with encapsulation efficiency and potential improvements in photostability. Finally, the strength of the drug-lipid interactions, as determined by FT-IR and UV-Vis, will be correlated with in vitro release studies to determine the impact of encapsulation on the release kinetics of PTX from the NLCs.

### Cytotoxicity evaluation

**Fig. 5:**
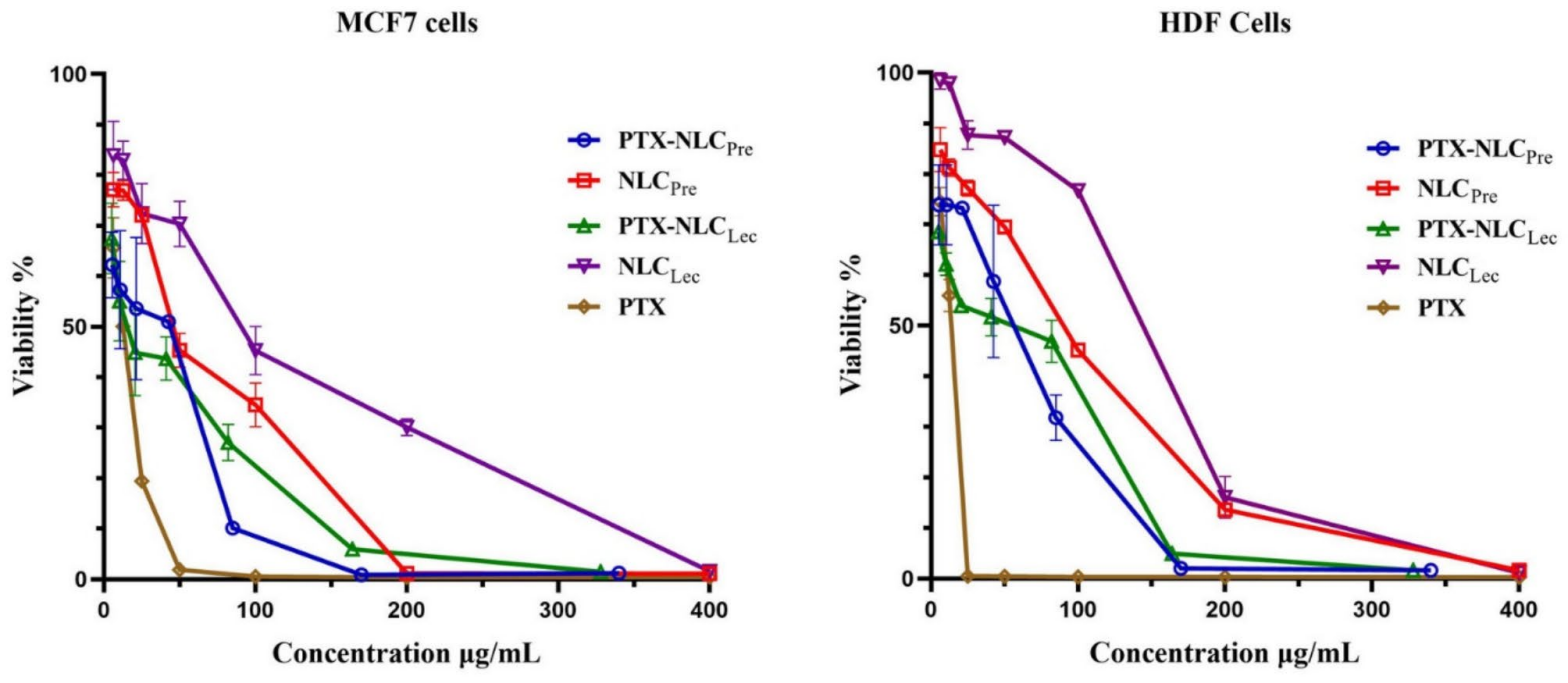
Cell viability of MCF-7 and HDF cells after treatment with PTX-loaded/unloaded NLCs and free PTX. The data, presented as percentage cell viability with standard deviation error bars, show the mean percentages from at least three independent experiments.

MTT assays on MCF-7 breast cancer and HDF normal fibroblast cell lines evaluated the cytotoxicity of free PTX versus PTX loaded within NLCs nanoparticles. Cell viability was determined via the MTT assay after 24 h of treatment. Absorbance readings were converted to percentage viability relative to untreated controls. Half-maximal inhibitory concentration (IC50) values were calculated using nonlinear regression in GraphPad Prism and defined as the drug concentration reducing viability by 50% (Fig. 5). As shown in Table 3, empty NLC_Lec_ nanoparticles elicited the lowest cytotoxicity against MCF-7 and HDF cells, with IC50 values exceeding 98 µg/mL. Commercial PTX demonstrated the highest cytotoxicity attributable to high Cremophor-EL content. No difference was evident between cell types for free PTX (*P* > 0.05). Viability decreased in a dose-dependent manner across treatments. Table 3 lists IC50 values, revealing the rank-order inhibition as: PTX > PTX-NLC_Lec_ > PTX-NLC_Pre_ > NLC_Pre_ > NLC_Lec_ for both cell lines. This profile was consistent between MCF-7 and HDF cells. No distinction was apparent between PTX-NLC_Pre_ and PTX-NLC_Lec_ (*P* > 0.05). However, the cytotoxicity of both loaded NLCs was significantly higher in MCF-7 versus HDF cells (*P* < 0.005), indicating selective cancer cell targeting. Notably, empty NLCs elicited lower cytotoxicity than loaded formulations (*P* < 0.005). While no difference emerged between NLC_Pre_ and NLC_Lec_ when loaded, empty NLC_Lec_ reduced viability to a lesser extent than NLC_Pre_ in both cell types (*P* < 0.0001). Overall, NLC_Lec_demonstrated minimal effects when empty but elevated killing when PTX-loaded. Comparison to our prior results^38^ revealed a cytotoxic rank-order of PTX-MF59 < PTX-NLC_Lec_ < PTX-NLC_Pre_ < MF59 < NLC_Pre_ < NLC_Lec_ for MCF-7 and HDF cells. MF59 significantly surpassed NLC_Lec_ cytotoxicity (*P* < 0.0001), indicating potential Lecithin-mediated attenuation. At higher treatment concentrations exceeding 50 µg/mL, empty NLC_Lec_ elicited the lowest cytotoxicity while free PTX induced the greatest reduction in cell survival across the tested dose range. Unloaded NLC_Pre_ also elicited lower viability than PTX-loaded formulations. Notably, no statistical difference was observed between PTX-MF59 and PTX- NLC_Lec_ regarding MCF-7 cell viability. The comparable effects observed further corroborate the conclusions that both nanoparticle systems effectively encapsulate and deliver PTX in a manner conferring anticancer activity.

It is important to highlight the significant advantages of our NLCs over Cremophor EL, particularly in addressing the known issues associated with its use.

Cremophor EL has been associated with cytotoxicity and hypersensitivity reactions, which can limit the maximum tolerated dose of PTX and complicate clinical applications^52^. In contrast, our NLC formulations demonstrated a favorable safety profile, with encapsulation efficiencies of 85% for NLC_Pre_−60 and 82% for NLC_Lec_−60, indicating robust drug loading without the adverse effects typically linked to Cremophor EL^53^.

**Table 3:** IC50 values of all treatment formulations in MCF-7 and HDF cells.

Nano-carrier type	Abbreviation name	IC50 for MCF-7 cells (µg/mL)	IC50 for HDF cells (µg/mL)
PTX-loaded NLC with Percirol	PTX-NLC_Pre_	27.17 ± 6.11	57.18 ± 16.92
Empty NLC with Percirol	NLC_Pre_	55.26 ± 5.72	90.57 ± 0.96
PTX-loaded NLC with Lecithin	PTX-NLC_Lec_	23.4 ± 3.4	54.11 ± 2.82
Empty NLC with Lecithin	NLC_Lec_	98.91 ± 11.44	128.50 ± 5.33
Free PTX Without Nano-carrier (contains Cremophor EL)	PTX	12.04 ± 1.33	12.90 ± 0.53

## Conclusion

Breast cancer represents a major global health burden, necessitating continued advancements in treatment efficacy and safety. PTX displays high potency against mammary tumors but suffers from solubility issues^54^ linked to toxicity when formulated with Cremophor EL.

This study builds upon our previous work evaluated MF59 nano-emulsion for PTX encapsulation and drug delivery system and confirmed its potential in breast cancer therapy. The formulated PTX-loaded MF59 was constructed using the homogenization method and exhibited higher cytotoxicity in MCF-7 cells compared to HDF cells^38^.

The significance of MF59 nano-mulsion in drug delivery systems is notable, particularly in the context of cancer therapy. MF59 has been administered to millions of individuals, establishing a strong safety profile with minimal reported toxicity. This is particularly relevant as we explore its application as a carrier for PTX. The inclusion of Squalene, a key component of MF59, enhances the stability of the formulation and possesses immune-stimulatory properties. Squalene has been shown to activate the immune system, which may provide an additional therapeutic benefit when used in conjunction with anticancer agents like PTX.

Here, we designed two novel PTX-loaded NLC compositions based on MF59 constituents to investigate as alternative cremophor-free formulations. Two PTX-NLC compositions incorporating Squalene, solid lipids, and MF59 additives were optimized for high drug EE, tunable particle characteristics, and controlled DR profiles. Physicochemical profiling and cytotoxicity evaluations demonstrated the optimized MF59-derived NLCs successfully encapsulated PTX while eliciting selective targeting of MCF-7 tumor cells over HDF normal fibroblasts. Our research addresses critical gaps in the stability, EE, and DR kinetics of PTX, leveraging the unique properties of MF59 and its immunogenic component, Squalene. This dual functionality presents a novel approach to cancer treatment, potentially enhancing therapeutic efficacy and immunogenic response.

Recent advancements in drug delivery systems, particularly those utilizing NLCs, have significantly contributed to enhancing the therapeutic efficacy of PTX in cancer treatment. For instance, Gao et al. developed nanoparticle albumin-bound PTX, demonstrating high drug loading and favorable pharmacokinetic profiles. Their study highlighted the importance of formulation parameters in achieving effective drug delivery^50^.

In another study, Majumder & Minko demonstrated the efficacy of multifunctional lipid-based nanoparticles for the co-delivery of anticancer drugs and siRNA, showcasing their potential in overcoming drug resistance in non-small cell lung cancer^55^.

In summary, our research demonstrates that NLCs provide a promising alternative to Cremophor EL for PTX delivery, effectively addressing critical issues of cytotoxicity and release kinetics while enhancing the overall therapeutic potential of the drug.

To further evaluate the therapeutic potential of the developed nano-carriers, we are currently conducting in vivo pharmacokinetic and anti-tumor efficacy studies using a mouse model of breast cancer. Preliminary results from these ongoing in vivo investigations will provide insight into the drug release behaviors and anti-cancer activities following administration. Overall, the results to date suggest MF59-based NLCs show promise as a new PTX delivery platform capable of enhancing solubilization, bioavailability, and safety profiles to maximize therapeutic outcomes. Continued preclinical assessment, including characterization of in vivo tumor responses, pharmacokinetics, and toxicity profiles, will help support the clinical translation of these cremophor-free nano-carriers.

## Data Availability

The data that support the findings of this study are available from the corresponding author upon reviewer request.

## Abbreviations

DR: Drug release
DL: Drug loadingp
DMEM: Dulbecco’s modified Eagle medium
DLS: Dynamic light scattering
EE: Encapsulation efficiency
FBS: Fetal bovine serum
FT-IR: Fourier-transform infrared
HDF: Human dermal fibroblasts
IC50: Inhibitory concentration
MCF-7: Michigan cancer foundation
MTT: 3-(4,5-dimethylthiazol-2-yl)-2,5-diphenyltetrazolium bromide
PTX: Paclitaxel
PDI: Polydispersity index
SEM: Scanning electron microscope
NLC: Nanostructured lipid carriers
UV-Vis: Ultraviolet-visible
